# Dispelling the myth: comparable duration and impact of research training for MD-PhD and PhD graduates

**DOI:** 10.1172/jci.insight.182288

**Published:** 2024-06-25

**Authors:** Rory Vu Mather, Temperance R. Rowell, Steve Obuchowski, Loren D. Walensky

**Affiliations:** 1 Harvard/MIT MD-PhD Program, Harvard Medical School, Boston, Massachusetts, USA.; 2 Department of Pediatric Oncology, Dana-Farber Cancer Institute, Boston, Massachusetts, USA.

## Abstract

The average time to degree for completing a life sciences PhD in the United States is longer for single-degree than dual-degree trainees, supporting a perception that the PhD training of MD-PhDs is less rigorous or fulsome. To determine whether degree format influences the duration and impact of graduate training, we analyzed data for the 2011–2016 graduates of 3 Harvard Medical School PhD programs. Linear mixed effects models were used to determine the association between degree type (MD-PhD vs. PhD) and research outcomes, including time to degree, time to thesis defense, and publications submitted during the PhD. Although pursuing an MD-PhD was associated with a 1.5-year shorter time to PhD degree, basing this calculation on the official PhD period does not account for completion of early PhD requirements, including research rotations and qualifying coursework, during the first 2 years of medical school. There was no association between degree format and total number of first-author or overall publications, though pursuing a dual degree was associated with increased impact metrics of published papers. The results highlight that despite the seemingly shorter PhD durations of MD-PhD graduates based on graduate program enrollment period, research training is on par with their single-degree peers, rendering MD-PhD graduates well equipped to become successful scientific investigators.

## Abstract 2

Despite optically shorter PhD timelines for MD-PhD compared to PhD graduates, the research training period and publication outputs are largely indistinguishable.

## Graphical abstract

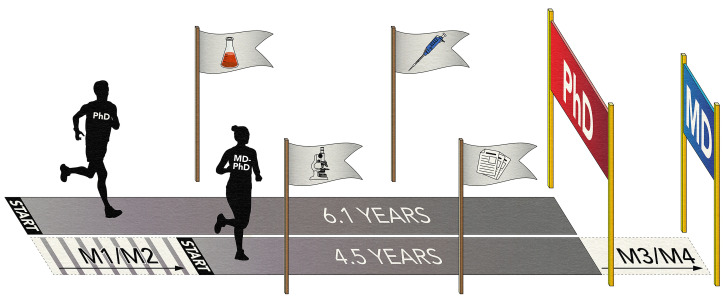

## Introduction

Dual-degree MD-PhD training programs were created as early as the late 1950s, with the goal of establishing a new workforce of physician-scientists who could bridge the gap between basic science and clinical practice (1). To master the skills required to practice medicine and conduct independent research, pursuing both degrees — generally involving 4 years of training each — would be required. Undertaking such lengthy times to degree was ultimately incentivized in 1964 by the NIH Medical Scientist Training Program (MSTP) that, in combination with institutional matching funds, covered the complete cost of training (2). By providing protected PhD research training time, MD students could tackle the full spectrum of basic and social science disciplines to advance the diagnosis, prevention, and treatment of human disease. Numerous studies have since documented the remarkable success of the MSTP initiative, with MD-PhD graduates having successful careers as reflected by academic positions, publications, and funding (3–8).

**Figure 1:**
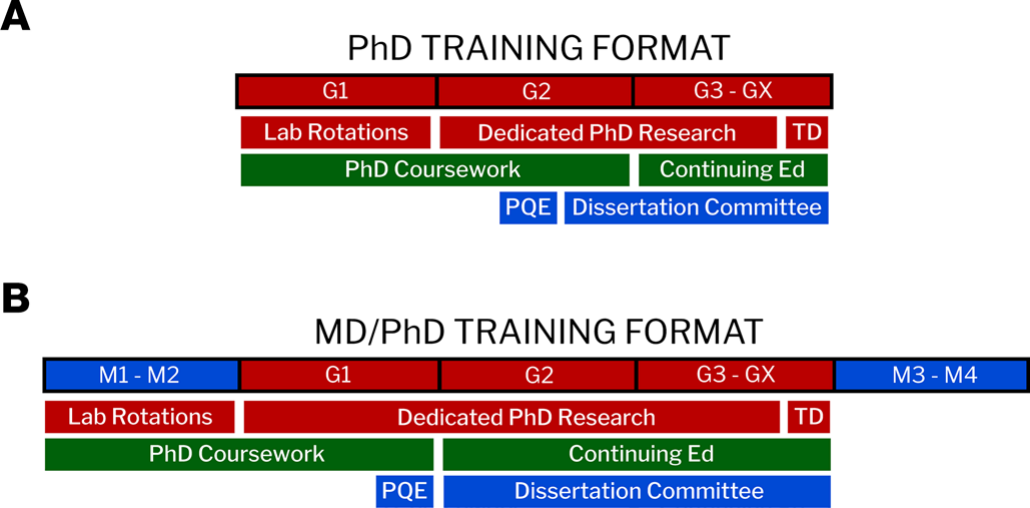
PhD training formats for single- and dual-degree students. (**A**) A PhD training program involves research, coursework (including professional development), and evaluation as distributed across the exemplary training timeline. (**B**) Key early components of PhD training are frameshifted for MD-PhD candidates, such that they overlap with years 1 and 2 of medical school (M1 and M2, respectively). TD, thesis defense; PQE, preliminary qualifying exam.

Although there are many similarities in the PhD training formats between single- and dual-degree programs, the combination with medical school courses offers MD-PhD programs a unique opportunity to eliminate overlap between the 2 degrees. PhD training programs are typically organized into 2 phases. In the first phase, students take a series of both required and elective graduate courses in the scientific field of focus, rotate in several laboratories to ultimately identify a PhD mentor and thesis project, serve as a teaching assistant, and prepare for the preliminary qualifying exam (PQE), which is designed to test the student’s acquisition of sufficient foundational knowledge to launch their dissertation research. Passing the PQE enables the student to progress to the intensive research phase of training, whereby the student predominantly focuses on thesis research, which culminates in the writing and defense of the PhD thesis, publications, and often abstract presentations at local, national, or international meetings (Figure 1A). Independent faculty committees are assembled to conduct the PQE examination, guide the PhD research via interval presentation and feedback meetings, and conduct the final dissertation examination.

MD-PhD programs follow a relatively standardized timeline whereby students start with 2 years of preclinical training along with introductory clinical rotation(s), transition to full-time graduate students, and then return to medical school after defending their PhD to complete years 3 and 4 of clinical training. Because there is often overlap between the preclinical coursework and that of the graduate program, MD-PhD students may be exempted from certain PhD program course requirements. In addition, MD-PhD students typically accomplish their laboratory rotations during the first 2 years of medical school, such that they have already identified a PhD mentor by the time they enter the PhD phase of training. Thus, it is noteworthy that, for MD-PhD students, certain time-consuming components of the first phase of PhD training are accomplished during the preclinical training period of medical school (Figure 1B).

The average time to graduation for MD-PhD programs nationally is 8.25 years, with 4.25 years dedicated to the PhD (3). A 2021 survey by the National Center for Science and Engineering Statistics of the National Science Foundation found that the average time to degree for a health sciences PhD was 8.8 years and for biological and biomedical sciences was 6.4 years (9). This dichotomy, along with efforts to streamline PhD training for MD-PhD students, has led to the question of whether PhD degrees in the context of MD-PhD training are less fulsome and impactful than those earned by single-degree candidates. To evaluate this hypothesis, we examined a series of performance metrics for PhD students who completed their graduate training in the Biological and Biomedical Sciences (BBS), Neuroscience, and Immunology programs at Harvard Medical School (HMS) as single- or dual-degree candidates between 2011 and 2016. Albeit a single-institution study, the large sizes of the PhD and MD-PhD student bodies not only allowed for sufficient numbers to achieve statistically significant comparisons but also ensured proper control for the graduate program curricula for single- and dual-degree cohorts. We find that although MD-PhD students finish their graduate phase of research training, formally defined by the period of official enrollment in graduate school, sooner than single-degree students, their productivity measured in number of publications and measures of research impact show either parity or better outcomes. These results highlight that the research training between dual- and single-degree students is largely indistinguishable such that MD-PhD students are well equipped to become successful scientific investigators.

## Results

### Student demographics.

**Figure 2:**
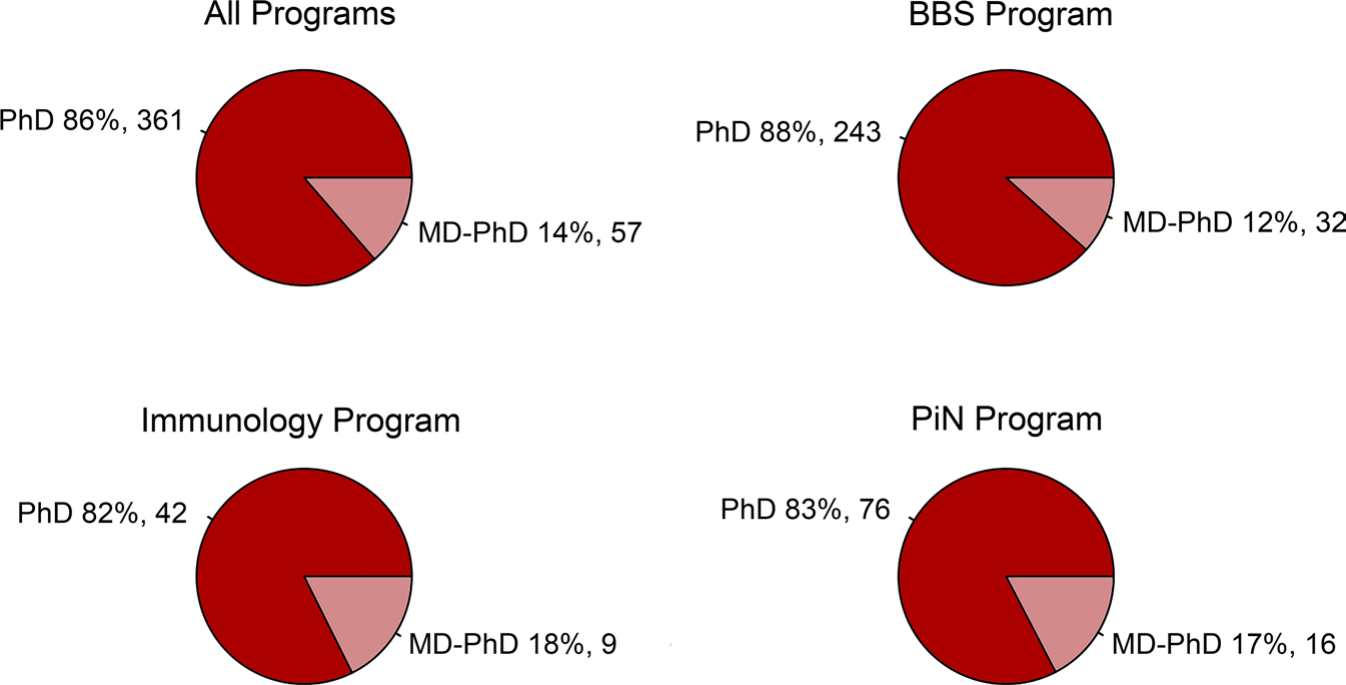
Percentage of students in each graduate program pursuing PhD or MD-PhD degrees. Across the 3 largest HMS graduate programs, single-degree PhD students account for 82%–88% and MD-PhD students represent 12%–18% of the overall graduate student body. BBS, Biological and Biomedical Sciences; PiN, Program in Neuroscience.

The BBS, Neuroscience, and Immunology programs of HMS graduated 507 PhD students between 2011 and 2016. Eighty-nine of these students had either incomplete demographic or publication data necessary for our linear models and were thus excluded from the final analyzed data set of 418 graduates (Supplemental Table 1; supplemental material available online with this article; https://doi.org/10.1172/jci.insight.182288DS1). Fifty-seven (13.6%) of the evaluated graduates were dual MD-PhD degree students, 233 (55.7%) were women, 34 (8.1%) identified as underrepresented in medicine (URM), and 88 (21.1%) were foreign students. The percentage of students graduating with a single or dual degree for each program is shown in Figure 2. The demographics and undergraduate grade point averages (GPAs) of each program, as grouped by PhD, MD-PhD, or all graduates, are listed in Table 1. When comparing the measured outcomes of PhD and MD-PhD students, we observed no significant differences based on the percentage of women in PhD (57.3%) compared with MD-PhD programs (45.6%) (*P* = 0.130), a lower proportion of URM students pursuing a PhD (6.9%) compared with MD-PhD (15.8%) degree (*P* = 0.044), and a higher percentage of foreign students pursuing a PhD (23.0%) compared with MD-PhD (8.8%) degree (*P* = 0.023) (Table 1). Since, for our linear models, the sensitivity analyses found that student sex, URM status, and foreign status did not lead to meaningful differences across analyses (Supplemental Figure 1), these variables were excluded from our models. We recognize, however, that there could be residual confounding from these variables not accounted for in our models. We further observed that there were no significant differences between degree groups with respect to overall undergraduate GPA (*P* = 0.053) or undergraduate major GPA (*P* = 0.166) and, within individual graduate programs, there were no significant differences in demographics or undergraduate GPAs between single- and dual-degree students (Table 1).

### Time to degree.

**Figure 3:**
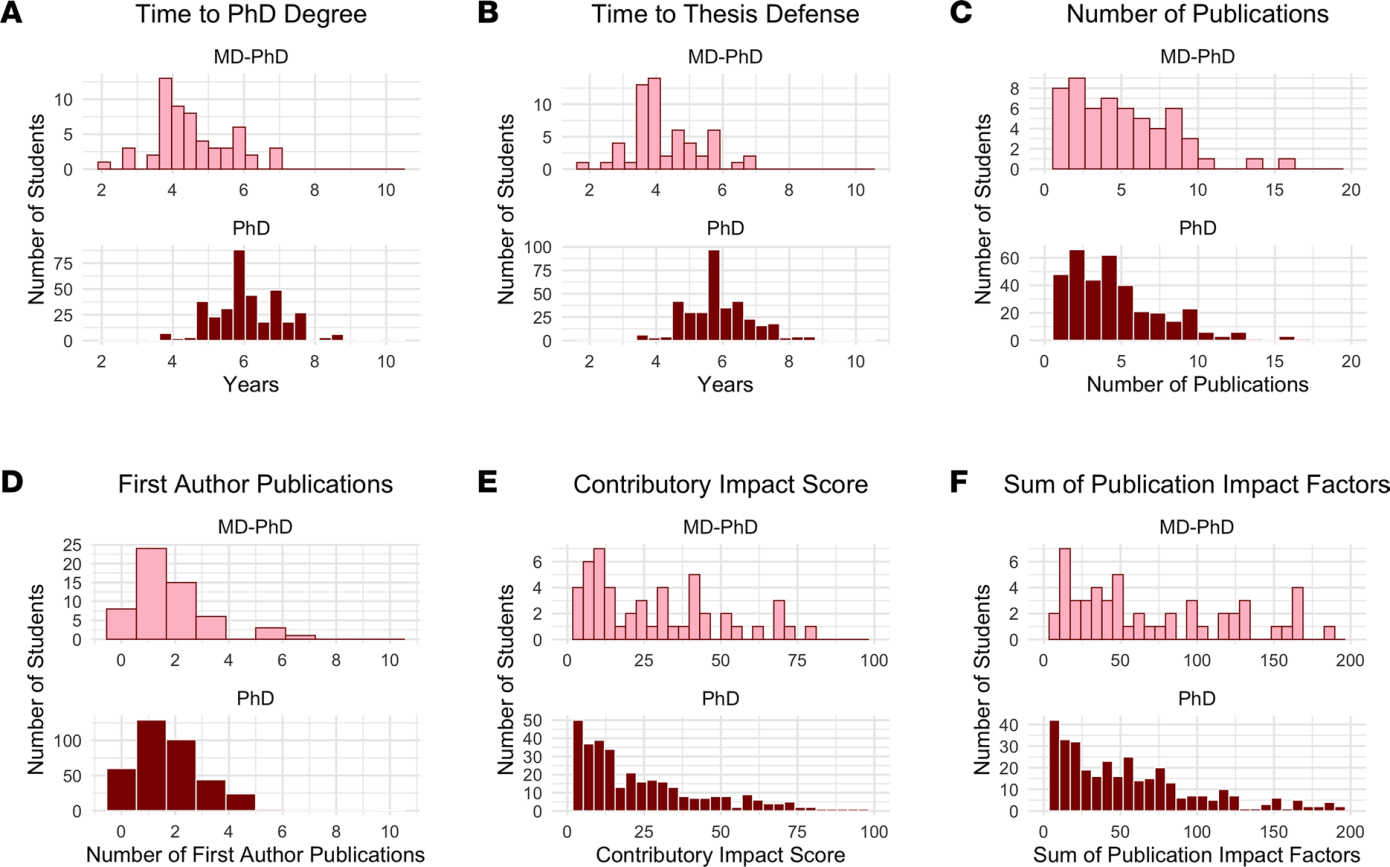
Distribution of training timeline and research outcomes across programs by single or dual degree. (**A**–**F**) Shown is the distribution of single PhD degree and dual MD-PhD degree students achieving a series of outcome metrics, including time to PhD degree (**A**), time to thesis defense (**B**), number of publications (**C**), number of first-author publications (**D**), contributory impact score (**E**), and sum of publication impact factors (**F**).

Within our cohort, MD-PhD students received their PhD degree in 4.5 years on average as compared with 6.1 years for their PhD counterparts (Figure 3A and Table 2), as calculated based on the official G1–GX PhD enrollment period (Figure 1). This shorter PhD timeline for MD-PhD students was apparent for each of the 3 graduate programs. After adjusting for potential confounders and accounting for variation between programs in our linear mixed effects model, we found that graduating with an MD-PhD was associated with a 1.5-year shorter time to PhD degree than graduating with a PhD alone (*P* < 2 × 10^–16^; 95% CI [1.2, 1.8]).

### Time to thesis defense.

MD-PhD students successfully defended their thesis dissertation in 4.3 years on average as compared with 5.9 years for PhD students (Figure 3B and Table 2), as calculated based on the official G1–GX PhD enrollment period (Figure 1). Across all PhD programs, the time to thesis defense was shorter for MD-PhD students than their PhD counterparts. After adjusting for potential confounders and accounting for variation between programs in our linear mixed effects model, we found that graduating with an MD-PhD was associated with a 1.6-year shorter time to thesis defense than graduating with a PhD (*P* < 2 × 10^–16^; 95% CI [1.3, 1.9]).

### Number of publications.

The average number of journal articles published based on graduate school research was similar for both MD-PhD (4.9 publications) and PhD students (4.7 publications) (Figure 3C and Table 2). After adjusting for potential confounders and accounting for variation between programs in our linear mixed effects model, we found that a single or dual degree was not a significant predictor (*P* = 0.600). The number of first-author publications was also similar between the 2 cohorts, with MD-PhD and PhD students having an average of 1.7 and 1.6 publications, respectively (Figure 3D and Table 2). Our linear mixed effects model showed that a single or dual degree was not associated with the number of first-author publications (*P* = 0.137).

### Contributory impact score.

To account for the relative contribution of the author to the published work, we developed a measure of research output, termed the “contributory impact score,” which takes the sum of journal impact factor divided by the authorship placement squared for each paper. This measure accounts for the impact factor of the publication but adjusts its significance based on the author’s contribution, such that, for example, a fourth authorship would be reflected by a decreased contributory impact score compared with a first authorship. MD-PhD students had an average contributory impact score of 34.7 as compared with 23.7 for PhD students (Figure 3E and Table 2). After adjusting for potential confounders and accounting for variation between programs in our linear mixed effects model, we found that dual MD-PhD degrees were associated with an 11.3 increase in score (*P* = 0.001; 95% CI [4.2, 18.3]). The average impact factor of the journals where MD-PhD students published their papers was 16.2 as compared with 13.5 for PhD students. A Wilcoxon rank sum test found that this difference was significant (*P* = 0.037).

We also examined whether the results of our publication analysis using our contributory impact score metric could be corroborated by a simplified measure of research output, namely the sum of the journal impact factors for each publication. MD-PhD students had an average sum of 83.7 as compared with 64.7 for PhD students (Figure 3F and Table 2). After adjusting for potential confounders and accounting for variation between programs in our linear mixed effects model, we found that dual MD-PhD degrees were associated with an increase of 20.2 in the sum of publication impact factors (*P* = 0.028); 95% CI [2.3, 38.2]), consistent with our contributory impact score findings.

## Discussion

In this study, we collected data from all graduates of the 3 largest HMS graduate programs from 2011 to 2016 to determine whether there are differences in the outcomes of research training, including time to degree, time to thesis defense, and publication output across single PhD degree and dual MD-PhD degree students. For both time to degree and time to thesis defense, we observed that the HMS student population follows the national trend of MD-PhD students graduating with shorter PhD timelines than their single PhD degree counterparts based on the graduate school enrollment period. Such data have led to the perception that MD-PhD students have streamlined PhD training. However, the respective 1.5-year and 1.6-year shorter time to degree and time to thesis defense demonstrated here for the 2011–2016 cohort of HMS MD-PhD students is readily accounted for by the accomplishment of a portion of PhD requirements during the first 2 years of medical school, prior to the official start of graduate school. Indeed, when research output is considered, with respect to the number and impact of publications, the 2 cohorts have a similar number of PhD-based publications (including first-author publications). If anything, the impact of the papers — whether measured by contributory impact score or sum of journal impact factors — is higher for dual-degree students.

Our results highlight that the research training MD-PhD and PhD students receive is largely indistinguishable. Whereas the standard training timeline for a PhD student includes coursework coupled with research rotations to identify a PhD mentor during G1 and G2, the majority of MD-PhD students receive graduate school credit for a portion of their M1–M2 coursework (that overlaps with PhD coursework) and pursue laboratory rotation research, typically in the afternoons during the semester or full-time during the summer between M1 and M2. Thus, MD-PhD students effectively initiate the dedicated PhD research period of the G1–GX PhD training format sooner than their single PhD degree peers, making the overall timeline largely similar. Indeed, our findings provide support and reassurance for recommending that MD-PhD programs incorporate early PhD requirements into the first and second years of medical school, including identifying coursework that fulfills dual requirements and avoids redundancy, holding exploratory meetings with candidate PhD mentors, and conducting research rotations when time allows. These approaches maximize training and progression, accounting for shorter PhD durations based on the official graduate program enrollment period, yet do not compromise research output or impact, as demonstrated by the cohort studied here.

The results and conclusions of our study derive from the analyses of PhD and MD-PhD program data from a single NIH MSTP–funded academic medical center, potentially limiting the generalizability of our findings. However, a key advantage of our study design is having the requisite size of a PhD and MD-PhD student community that allows for statistically significant conclusions to be drawn while avoiding the confounder of heterogeneity across institutions in individual graduate training curricula. Here, the PhD and MD-PhD student cohorts are subject to the same graduate school environment and requirements. We intentionally limited our outcome measure analyses to metrics that can be directly linked to graduate training, such as training timeline and graduate-phase publications, rather than longer term metrics, such as success rates in garnering postdoctoral fellowships or junior faculty grants. This is because the latter are confounded by training influences beyond the graduate phase and require studying older cohorts whose graduate training environment was more distant from the current educational climate, such as the more recent trend of PhD and MD-PhD students to take gap years prior to application and matriculation (10).

Our findings of parity in graduate school outcomes are consistent with longer term outcomes reported in other studies, such as the Physician-Scientist Workforce Working Group Report, which was published in 2014 (2). In this study, MD-PhD and PhD investigators were found to have similar success rates in obtaining research funding. In examining the percentage of applicants who were awarded an NIH Research Project Grant (RPG) in 2012, 24.6% of MD-PhD applicants were successful as compared to 21.7% of PhD applicants. The difference was likewise only marginal when considering R01 application success rates (20% for MD-PhD compared to 19% for PhD). Data collected by the National Science Foundation and NIH have shown that MD-PhD graduates remain in academia at higher rates (67%) compared with PhD graduates (47.5% of health sciences PhDs and 22.6% of biology and biomedical sciences PhDs) (9, 11). Although MD-PhD graduates currently make up less than 2% of all PhD graduates, dual-degree investigators account for 14.8% of awarded NIH RPGs (8, 12). Taken together, these results indicate that the rigor and impact of the PhD phase of MD-PhD training match that of single-degree programs, yielding physician-scientist graduates who are well equipped to become successful independent investigators.

## Methods

### Data sources.

We collected data for the 2011–2016 graduates of 3 HMS PhD programs, including the BBS program, PiN, and Immunology program. This study time frame was selected to both enable sufficient time from degree conferral to achieve the publication of PhD research and avoid confounding effects of the COVID-19 pandemic in delaying graduation and publication timelines. The study cohort of MD-PhD graduates trained during a period where no curriculum changes occurred in the M1–M2 phase. Student demographic data, including sex, URM status, and foreign student status, and overall and undergraduate major GPAs were anonymized, compiled, and included in our models as adjustment variables. Sensitivity analyses were performed on these variables to determine if they meaningfully impacted outcomes and thus warranted inclusion in our model. The outcome measures collected and subjected to analysis were time to degree (defined from the official start of the PhD program to degree conferral), time to thesis defense (defined from the official start of the PhD program to the date of successful thesis defense), and the roster of student publications submitted during the PhD along with the student’s authorship placement. We chose to analyze both time to degree and time to thesis defense because degree conferral occurs only at a select number of dates annually and therefore may not accurately represent the time spent performing research contributing to the completion of the PhD degree. Of note, MD-PhD students are required to submit and defend their PhD thesis prior to returning to M3, and the overall MD training phase is 4 years. For students who took a leave of absence during their graduate degree, this time was not included when calculating their time to degree or time to thesis defense. Student publications from the PhD training period were queried in January 2021, which is at minimum 4 years after each student’s degree conferral and should thus capture most if not all papers submitted during the PhD. As an added component of our analysis, we created a measure of research output termed the “contributory impact score,” which takes into account the sum of the journal impact factor for each publication divided by author placement squared. We used journal impact factors published as of 2019 to calculate the score (13). This measure aims to account for not only the impact of each publication on the scientific community as measured by the journal’s impact factor but also the relative contribution of the PhD student to the published work as reflected by author placement. We also calculated the sum of the impact factors of the journals for each publication as an alternative, simplified measure of research output.

### Statistics.

The proportions of female, URM, and foreign students in single- and dual-degree programs were compared using χ^2^ tests and Fisher exact tests when the number of individuals with analytic subgroups was below 5 individuals. Undergraduate GPA was summarized as means and SDs by degree format. Differences in group means were tested with 2-tailed, unpaired Student’s *t* tests. To determine the association between degree type (MD-PhD vs. PhD) and research outcomes, we built linear mixed effects models. The PhD program (BBS, PiN, Immunology) was included as a random effect to account for variation in outcomes between programs. Overall undergraduate GPA was included as an adjustment variable for potential confounding. For our models predicting time to degree and time to thesis defense, we included the number of publications as an adjustment variable. For our model predicting number of publications, we included time to degree as an adjustment variable. Adjustment variables were included as covariates in each model to account for potential confounding and, therefore, were not interpreted. All analyses were completed in R (version 4.2.1). A *P* value less than 0.05 was considered significant.

### Study approval.

The study was deemed not to be human subject research in accordance with the Harvard Longwood Campus Institutional Review Board Decision Tool. All data were reported in aggregate or deidentified.

### Data availability.

Deidentified data for this study can be found in the Supporting Data Values file, Supplemental Table 1, and Supplemental Figure 1.

## Author contributions

RVM, TRR, and LDW designed the study. SO acquired data. RVM, TRR, and LDW performed data analysis. RVM, TRR, SO, and LDW prepared the manuscript. LDW supervised the project.

## Supplementary Material

Supplemental data

Supporting data values

## Figures and Tables

**Table 2:**
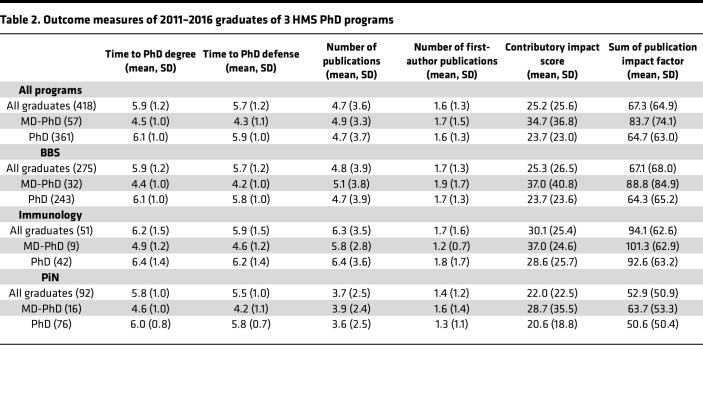
Outcome measures of 2011–2016 graduates of 3 HMS PhD programs

**Table 1:**
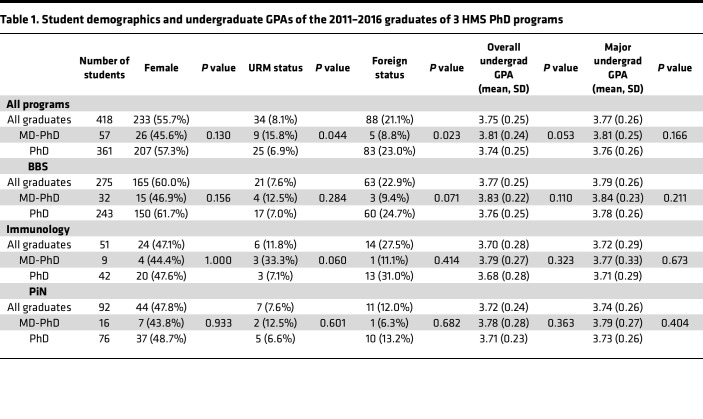
Student demographics and undergraduate GPAs of the 2011–2016 graduates of 3 HMS PhD programs

## Version 1 06/25/2024

In-Press Preview

## Version 2 08/08/2024

Electronic publication
