# Supplementary material for: Dispelling the myth: comparable duration and impact of research training for MD-PhD and PhD graduates
Source: JCI Insight. 2024 Jun 25;9(15):e182288. doi: 10.1172/jci.insight.182288 (PMC11383590; doi:10.1172/jci.insight.182288)
Supplement: Supplemental data [file jciinsight-9-182288-s006.pdf]

Supplemental Table 1. Distribution of samples excluded due to incomplete data.

	Number of Samples Excluded
**All Programs**	
All Graduates	89
MD-PhD	5
PhD	84
**Biology and Biological Sciences**	
All Graduates	61
MD-PhD	4
PhD	57
**Immunology**	
All Graduates	11
MD-PhD	1
PhD	10
**Program in Neuroscience**	
All Graduates	17
MD-PhD	0
PhD	17

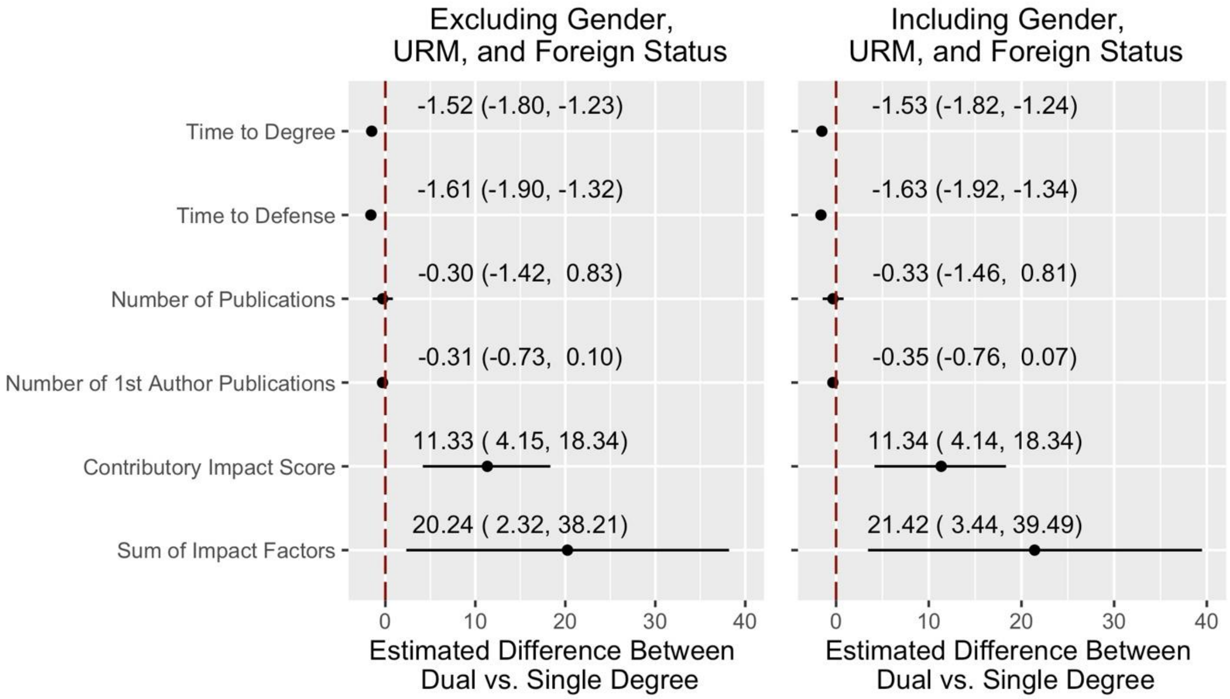

Supplemental Figure 1. Forest plots of model outcomes compared to the model adjusting for gender, URM, and foreign student statuses. Each line of the forest plot represents the estimated difference with confidence interval for each outcome when comparing dual and single degree students. The left panel presents the estimates for the model outcomes that do not include gender, URM, or foreign student statuses as adjustments. The right panel presents the estimates for the model that does include gender, URM, and foreign student statuses as adjustments. The dashed red line represents the null hypothesis of no difference in outcome.
